# Supplementary figures and images for: A global, regional, and national survey on burden and Quality of Care Index (QCI) of bladder cancer: The global burden of disease study 1990–2019
Source: PLoS One. 2022 Oct 20;17(10):e0275574. doi: 10.1371/journal.pone.0275574 (PMC9584505; doi:10.1371/journal.pone.0275574)

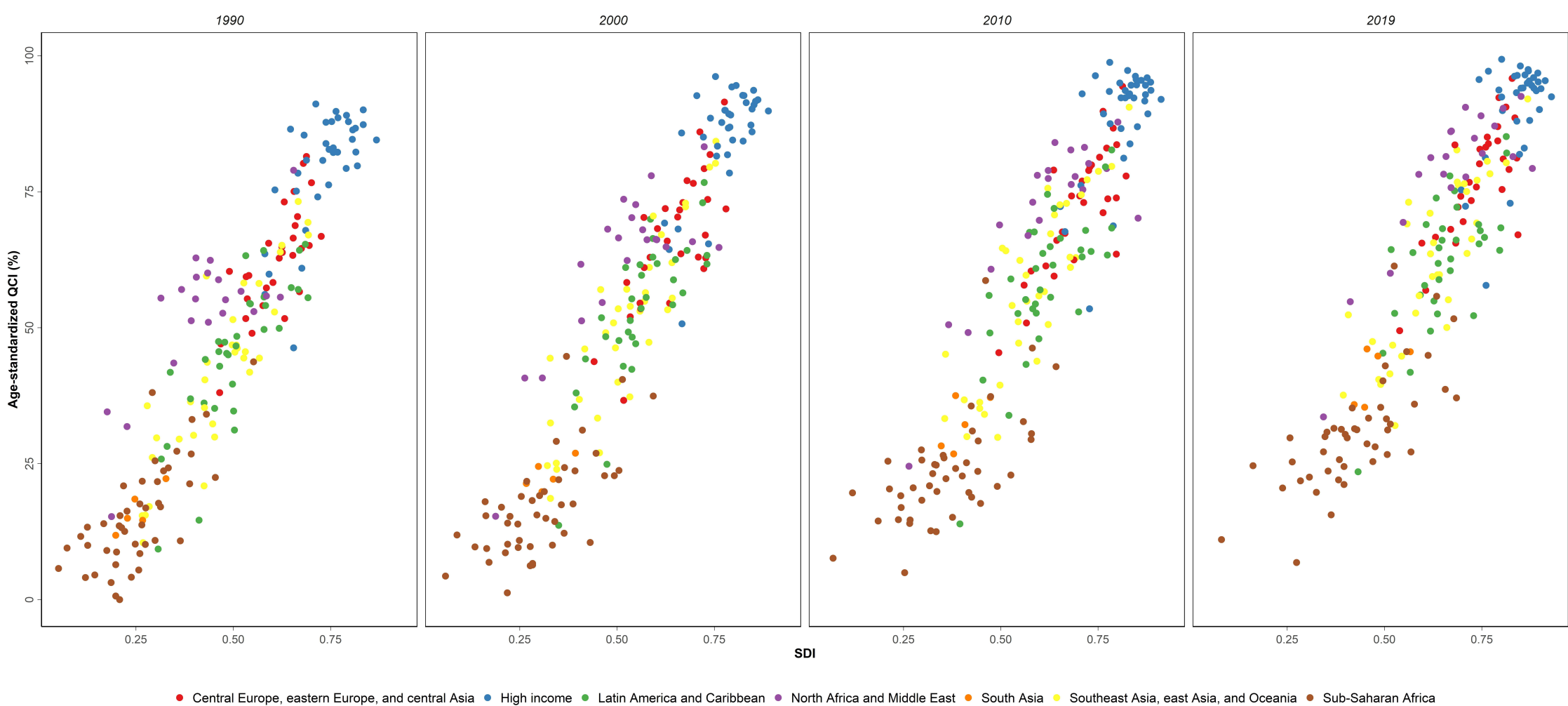

Supplement: S1 Fig — (PDF) [file pone.0275574.s001.pdf]
