# Supplementary material for: A global, regional, and national survey on burden and Quality of Care Index (QCI) of bladder cancer: The global burden of disease study 1990–2019
Source: PLoS One. 2022 Oct 20;17(10):e0275574. doi: 10.1371/journal.pone.0275574 (PMC9584505; doi:10.1371/journal.pone.0275574)
Supplement: S1 Table — (PDF) [file pone.0275574.s002.pdf]

ICD-10 code	Described Term
C67	Malignant neoplasm of bladder
C67.0	Malignant neoplasm of trigone of bladder
C67.1	Malignant neoplasm of dome of bladder
C67.2	Malignant neoplasm of lateral wall of bladder
C67.3	Malignant neoplasm of anterior wall of bladder
C67.4	Malignant neoplasm of posterior wall of bladder
C67.5	Malignant neoplasm of bladder neck
C67.6	Malignant neoplasm of ureteric orifice
C67.7	Malignant neoplasm of urachus
C67.8	Malignant neoplasm of overlapping sites of bladder
C67.9	Malignant neoplasm of bladder, unspecified
D09.0	Carcinoma in situ of bladder
D30.3	Benign neoplasm of bladder
D41.4	Neoplasm of uncertain behavior of bladder
D41.8	Neoplasm of uncertain behavior of other specified urinary organs
D49.4	Neoplasm of unspecified behavior of bladder
Z12.6	Encounter for screening for malignant neoplasm of bladder
Z12.7	Encounter for screening for malignant neoplasm of other genitourinary organs
Z12.79	Encounter for screening for malignant neoplasm of other genitourinary organs
Z80.52	Family history of malignant neoplasm of bladder
Z85.51	Personal history of malignant neoplasm of bladder
