# Supplementary material for: A global, regional, and national survey on burden and Quality of Care Index (QCI) of bladder cancer: The global burden of disease study 1990–2019
Source: PLoS One. 2022 Oct 20;17(10):e0275574. doi: 10.1371/journal.pone.0275574 (PMC9584505; doi:10.1371/journal.pone.0275574)
Supplement: S2 Table — (DOCX) [file pone.0275574.s003.docx]

**S2 Table:** The coefficients of the mixed-effect regression model

| Variable | Coef. | Std. Err. | P>\|z\| | [95% Conf. Interval] | |
| --- | --- | --- | --- | --- | --- |
|  |  |  |  | Lower | Upper |
| Inpatient care utilization | 2.00 | 5.75 | 0.727 | -9.26 | 13.27 |
| outpatient care utilization | 2.84 | 0.14 | <0.001 | 2.58 | 3.11 |
| BCa Prevalence | 4.17 | 0.74 | <0.001 | 2.73 | 5.62 |
| BCa Deaths | -8.32 | 0.36 | <0.001 | -9.03 | -7.60 |
| attributed death to BCa risk factors | 0.59 | 0.01 | <0.001 | 0.57 | 0.61 |
| constant | 44.31 | 1.46 | <0.001 | 41.45 | 47.16 |

BCa: Bladder cancer
