# Supplementary material for: A global, regional, and national survey on burden and Quality of Care Index (QCI) of bladder cancer: The global burden of disease study 1990–2019
Source: PLoS One. 2022 Oct 20;17(10):e0275574. doi: 10.1371/journal.pone.0275574 (PMC9584505; doi:10.1371/journal.pone.0275574)
Supplement: Attachment — Submitted filename: Response_2022.08.24.docx [file pone.0275574.s004.docx]

Regarding Figure 1, was any software used in the creation of the figure? If so, please tell us which software and provide us with any relevant links and/or copyright information.

As we had mentioned in the method section of the manuscript, all the statistical analyses and illustrations were carried out using R statistical packages v4.0.4 (http://www.r-project.org/, RRID: SCR_001905). The maps in figure 1 were created using Open Street Map’s data (without the use of an Open Street Map tile), and the Open Street Map’s shapefile was used in R statistical software to prepare these figures. To respond to your valuable inquiry, we revised the figure caption in the submitted files as “***Contains information from OpenStreetMap and OpenStreetMap Foundation, which is made available under the Open Database License***”.
